# Introduction of 7‐day amotosalen/ultraviolet A light pathogen‐reduced platelets in Honduras: Impact on platelet availability in a lower middle‐income country

**DOI:** 10.1111/vox.13740

**Published:** 2024-10-07

**Authors:** Marcelo Pedraza, Julio Mejia, John P. Pitman, Glenda Arriaga

**Affiliations:** ^1^ Programa Nacional de Sangre Cruz Roja Hondureña (Honduran Red Cross [HRC]) Tegucigalpa Honduras; ^2^ Scientific and Medical Affairs Cerus Corporation Concord California USA

**Keywords:** amotosalen, Honduras, pathogen reduction, platelets

## Abstract

**Background and Objectives:**

Honduras became the first lower middle‐income country (LMIC) to adopt amotosalen/UVA pathogen‐reduced (PR) platelet concentrates (PCs) as a national platelet safety measure in 2018. The Honduran Red Cross (HRC) produces ~70% of the national platelet supply using the platelet‐rich plasma (PRP) method. Between 2015 and 2018, PCs were screened with bacterial culture and issued as individual, non‐pooled PRP units with weight‐based dosing and 5‐day shelf‐life. PR PCs were produced in six‐PRP pools with a standardized dose (≥3.0 × 10^11^), no bacterial screening and 7‐day shelf‐life. Gamma irradiation and leukoreduction were not used.

**Materials and Methods:**

PC production and distribution data were retrospectively analysed in two periods. Period 1 (P1) included 3 years of PRP PCs and a transition year (2015–18). Period 2 (P2) included 5 years of PR PCs (2019–23). PC doses were standardized to an equivalent adult dose for both periods. Descriptive statistics were calculated.

**Results:**

HRC produced 10% more PC doses per year on average in P2 compared to P1. Mean annual waste at HRC declined from 23.9% in P1 to 1.1% in P2. Two urban regions consumed 96% of PC doses in P1 and 88.3% in P2. PC distributions increased in 14/18 regions.

**Conclusion:**

Standardized dosage, PR and 7‐day shelf‐life increased PC availability, reduced waste, eliminated bacterial screening and avoided additional costs for arboviral testing, leukoreduction and irradiation. Access to PC transfusion remains limited in Honduras; however, the conversion to pooled PR PCs illustrates the potential to sustainably expand PC distribution in an LMIC.

**Highlights**

- Amotosalen/UVA pathogen‐reduced (PR) platelet concentrates (PCs) have been used sustainably as the national standard in Honduras since 2018.
- Standardized pooling and dosing reduced PC waste.
- The 7‐day shelf‐life of PR PCs allowed expanded distribution of platelets to rural areas of a lower middle‐income country.

## INTRODUCTION

Platelet transfusion is an important supportive therapy for thrombocytopenic patients, including haematology/oncology patients, to prevent or treat bleeding associated with various chemotherapies, organ transplantation and haematopoietic stem‐cell transplantation (HSCT); trauma patients with active haemorrhage or patients with bleeding disorders [1]. Platelets are collected and transfused worldwide, however, platelet supplies vary dramatically between countries, most notably between upper income and lower middle income countries (LMIC) [2]. The World Bank defines LMIC as countries with per capita gross national income (GNI) between $1136 and $4465 [3].

**FIGURE 1:**
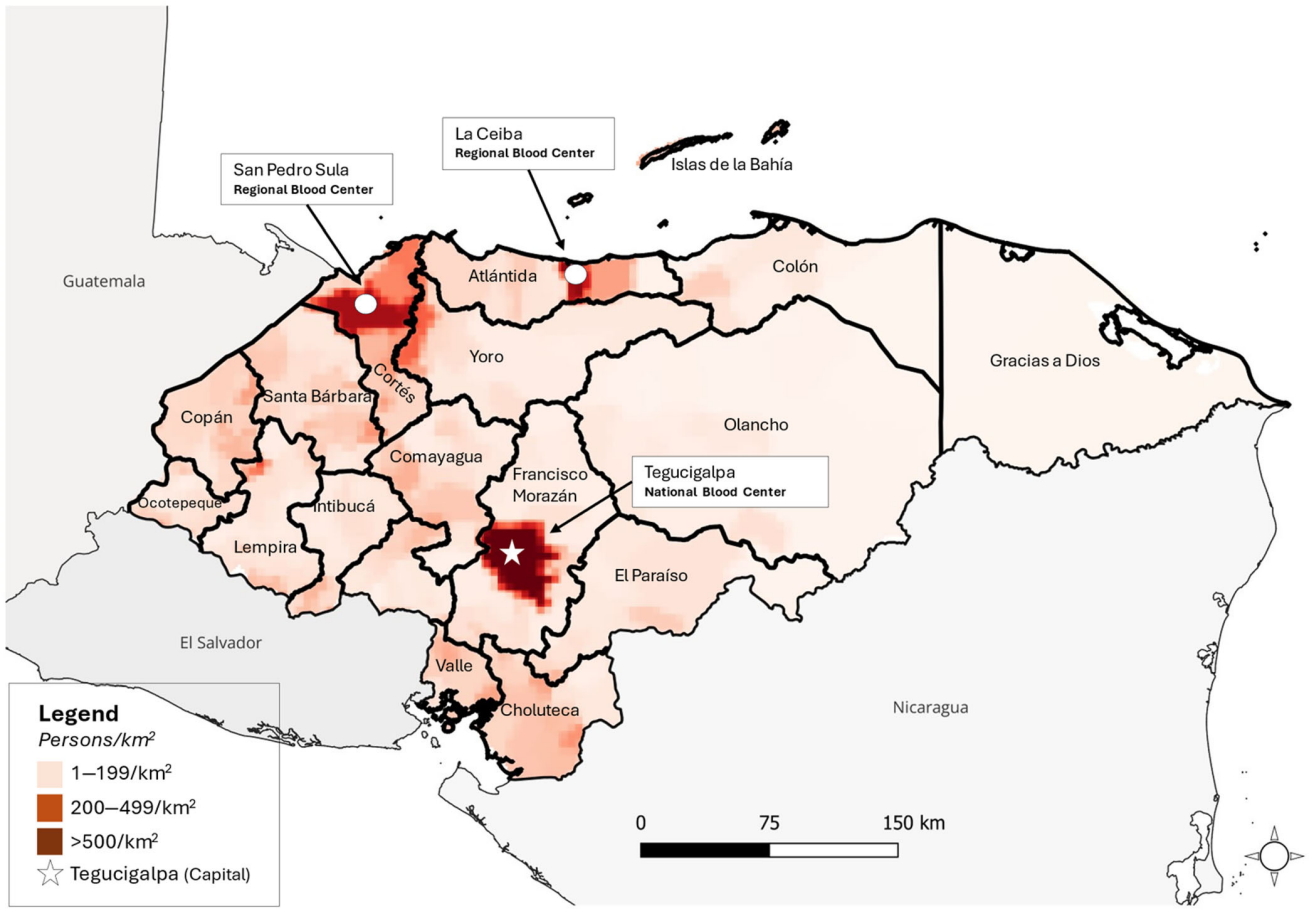
Geographic distribution of Honduran population expressed as persons per square kilometre; identification of high and low population areas and locations of Red Cross blood centres in relation to population centres.

Honduras is an LMIC with a population of approximately 10 million people in Central America. Approximately half of the population resides in three large urban areas, including the capital city, Tegucigalpa (Figure 1).

A number of transfusion‐transmissible arboviral and other vector‐borne diseases are endemic to Honduras, including dengue virus, chikungunya virus and Chagas disease [4, 5]. Data on arboviral prevalence in Honduran blood donors are not available; however, arboviral risk in blood donors has been established in endemic areas elsewhere in Latin America and the Caribbean [6, 7, 8]. Other blood‐borne pathogens, including human immunodeficiency virus (HIV), hepatitis B and C viruses (HBV, HCV), human T‐lymphotropic virus (HTLV) and syphilis have been documented in Honduran blood donors at relatively high levels. A serosurvey of Honduran blood donors conducted between 2014 and 2016 found that >2% of 48,567 donors were infected with a transfusion‐transmissible infection (TTI) [9]. Bacterial contamination of platelets is not widely documented in Latin America due to limited haemovigilance [10]; however, hospital‐based haemovigilance systems have documented transfusion‐transmitted bacterial infections (TTBIs) in Brazil and Colombia [11, 12]. A 2021 review estimated that actual TTBI rates in Latin American countries could be 7–29 times higher than reported estimates [13].

The Honduran Red Cross (HRC) is responsible for collecting, preparing and distributing ~70% of the national blood supply, including platelets. The balance of labile blood products are produced by hospital blood banks. HRC is reimbursed for blood components transfused in public hospitals from governmental budgets assigned to the Secretary of Health and the Secretary for Social Security, which operate the national network of public hospitals. Out‐of‐pocket reimbursements are paid by patients transfused in the private sector.

HRC operates three blood centres: The National Blood Centre in Tegucigalpa and Regional Blood Centres in La Ceiba (Atlántida region) and San Pedro Sula (Cortés region) (Figure 1). HRC collects >47,000 whole blood donations per year, mostly (>70%) from family‐replacement donors [2, 9]. Two of the three HRC blood centres (Tegucigalpa and San Pedro Sula) have been accredited by the Association for the Advancement of Blood and Biotherapies (AABB) since 2000. A small number of apheresis platelets are produced each year; the majority of the national platelet supply is produced from platelet‐rich plasma (PRP). Before the introduction of pathogen reduction, platelets were produced as individual PRP units (PRPs), which were prescribed using a weight‐based formula.

Leukoreduction and gamma irradiation were not used for platelet concentrate (PC) production in Honduras during the entire study period.

All whole blood donations are screened for HIV, HBV and HCV with an electro‐chemiluminescence assay (ECLIA). To reduce the risk of window period infections, HRC uses nucleic acid testing (NAT) to re‐screen donations with a negative ECLIA result. Serological assays are also used to screen donations for syphilis, HTLV‐1 and 2 and hepatitis B core antigen. HRC introduced bacterial culture screening with the BacT/Alert system (bioMérieux, Marcy l'Étoile, France) for all platelets in 2015. Aerobic bottles were inoculated with a 7–10 mL sample between 12 and 24 h post‐collection and incubated for at least 12 h prior to release. In 2016, challenges with reagent procurement and concerns about emerging and other non‐bacterial infectious risks such as Zika virus [14] led the HRC to evaluate the amotosalen/UVA pathogen reduction technology (INTERCEPT® Blood System, Cerus Corporation, Concord, California, USA) as an enhanced safety measure for platelets. The HRC implemented amotosalen/UVA pathogen reduction for 100% of the PCs produced by HRC blood centres in 2018. This article describes the Honduran experience as the first LMIC to implement pathogen‐reduced (PR) PCs at a national scale and sustain the practice for 5 years.

## MATERIALS AND METHODS

**FIGURE 2:**
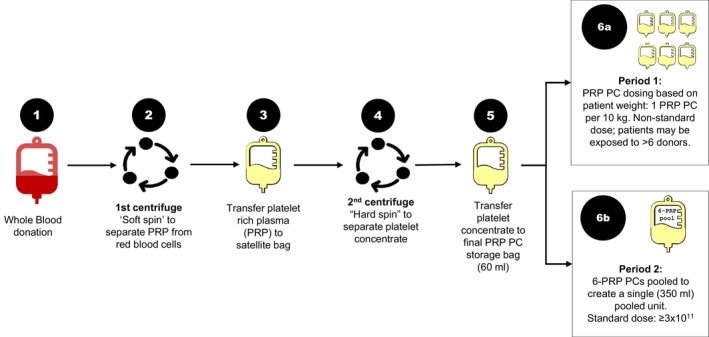
Procedure used to produce platelet components in Honduras: The platelet‐rich plasma (PRP) method. PC, platelet concentrate.

Whole blood collections, PC production and distribution data were retrospectively extracted from HRC databases and stratified by year, region and type of PC. Data were further stratified into 2 discrete time periods. Period 1 (P1) covered 3 years of conventional PRP PC production (2015–2017) plus the transition year (2018). Period 2 (P2) covered the first 5 complete years of pooled PR PC production and distribution (2019–2023). PC doses were standardized to an equivalent adult dose for both periods: PRP doses in P1 were based on a 1 PRP per 10 kg dosing formula and a mean adult (male and female) weight estimate of 68 kg [15]. Pooled PR PC doses produced in P2 were prepared with six non‐leukoreduced, non‐irradiated PRPs and a standardized target dose of ≥3.0 × 10^11^ (Figure 2).

HRC conducted a validation study in 2018 to assess the quality of PCs prepared and stored with the INTERCEPT Blood System for Platelets. The study measured PC volume, platelet concentration (×10^9^/L), platelet dose (×10^11^) and pH through storage day 7.

A cost–benefit analysis was performed based on the model of anticipated savings associated with pathogen reduction technology described by McCullough et al. [16]. Baseline costs included the use of a quadruple bag collection system for PRP production, consumables for bacterial culture screening and estimated costs associated with false positive results. Pathogen reduction costs assumed the use of a triple bag collection system with pooling sets and consumables, for example, sterile docking seals; the elimination of bacterial screening; the use of INTERCEPT large volume (LV) processing sets and savings associated with reduced processing and transfusion reaction costs. Additional savings included the avoidance of implementing gamma irradiation to prevent transfusion‐associated graft‐versus‐host disease (TA‐GVHD) and leukoreduction to reduce the risk of cytomegalovirus (CMV) infection, alloimmunization and clinical refractoriness [17]. Future cost‐savings associated with avoided future testing costs were also estimated. All costs (in US Dollars) were based on actual 2017 prices and estimated prices listed by McCullough et al.

Population estimates (national and for 18 regions) were derived from the Honduran National Institute of Statistics [18]. Descriptive statistics (means, ranges) were calculated for two‐period comparisons and multi‐year trends. No statistical testing was performed. Maps were produced with QGIS version 3.36.1 (QGIS.org).

## RESULTS

A total of 28,449 equivalent PRP PC doses were produced during the 9‐year study period (11,865, 41.7% in P1; 16,584, 58.3% in P2). On average, 2996 PC doses were produced annually in P1 versus 3317 produced per year in P2 (+11.8%). The proportion of PC doses distributed for transfusion increased from 64.6% (1844 / 2854) in 2015 when shelf‐life was limited to 5 days (>35% waste) to 98.8% (3126 / 3164) in 2023 after the extension of shelf‐life to 7 days (~1.2% waste). Annual PC waste at HRC decreased from 23.9% per year on average in P1 to 1.1% per year in P2 (Table 1). The majority of expirations in P1 occurred at the HRC production centre before release. Most expirations in P2 occurred at the receiving hospital after distribution from the HRC production centre. Platelets were released on the afternoon of Day 1 post‐collection on average in both periods (Figure 4).

**TABLE 1:** Nine‐year summary of PC production, distribution and waste, Honduran Red Cross, 2015–2023.

		Individual PRPs + 2018 transition (P1)				Pooled amotosalen/UVA PCs (P2)				
		2015	2016	2017	2018^a^	2019	2020	2021	2022	2023
PC doses produced	*N*	2854	3283	2910	2818	3316	2826	3914	3364	3164
	Mean	2966				3317				
PC doses distributed	*N*	1844	2223	2166	2752	3278	2791	3884	3330	3126
	Mean	2246				3282				
Estimated waste (%)	%	35.4%	32.3%	25.6%	2.3%	1.1%	1.2%	0.8%	1.0%	1.2%
	Mean	23.9%				1.1%				
% PR		0%	0%	0%	~10%	100%	100%	100%	100%	100%
Platelet shelf‐life (days)		5			5/7	7				

Abbreviations: PC, platelet concentrate; PR, pathogen‐reduced; PRP, platelet‐rich plasma.

^a^ Pathogen reduction transition year. The grey shading is meant to highlight 2018 as the year HRC transitioned to pathogen reduction, that is, in this year the data reflect ~10% PR and 90% conventional, vs. 100% of conventional in 2015‐2017 and 100% PR in 2019‐2023.

No cases of TA‐GVHD were documented in either period.

Whole blood collections increased by 49% in 2023 compared with 2015. This increase was driven in large measure by overall population growth between 2015 and 2023. On a national basis, mean annual whole blood collections (~450 mL) did not change substantially when factored against population growth: 4.0 whole blood (WB) collections per 1000 population in P1 versus 4.4 per 1000 population in P2 (Table 2).

**TABLE 2:** National RBC and PC production by population.

	Period 1 (P1)				Period 2 (P2)				
	2015	2016	2017	2018	2019	2020	2021	2022	2023
Population (pop.)	8,574,532	8,701,014	8,866,351	9,012,229	9,158,345	9,304,380	9,450,711	9,597,042	9,743,373
RBCs^a^	32,055	33,347	36,332	38,267	41,103	30,354	41,593	46,733	47,956
PC doses	2854	3283	2910	2818	3316	2826	3914	3364	3164
RBC per 1000 population (pop.)	3.7	3.8	4.1	4.2	4.5	3.3	4.4	4.9	4.9
PC per 1000 pop.	0.33	0.38	0.33	0.31	0.36	0.30	0.41	0.35	0.32
Mean PC/1000 pop.	0.23				0.34				
Mean RBC/1000 pop.	4.0				4.4				

Abbreviation: PC, platelet concentrate.

^a^ Red blood cells (RBCs) are used as a proxy for whole blood (WB) collections as 97%–99% of WB collections were processed into RBCs each year.

### Population growth and geographic distribution of PC doses

The national population of Honduras grew 13.7% between 2015 and 2023, increasing from approximately 8,574,532 in 2015 to 9,743,373 in 2023, according to estimates derived from the 2013 Honduran Census [18]. Annual population growth by region averaged 1.6% (range: 1.2%–2.5%). Urban areas in three regions (Francisco Morazán, Cortés and Atlántida) accounted for ~42% of the national population throughout the 9‐year study period.

**FIGURE 3:**
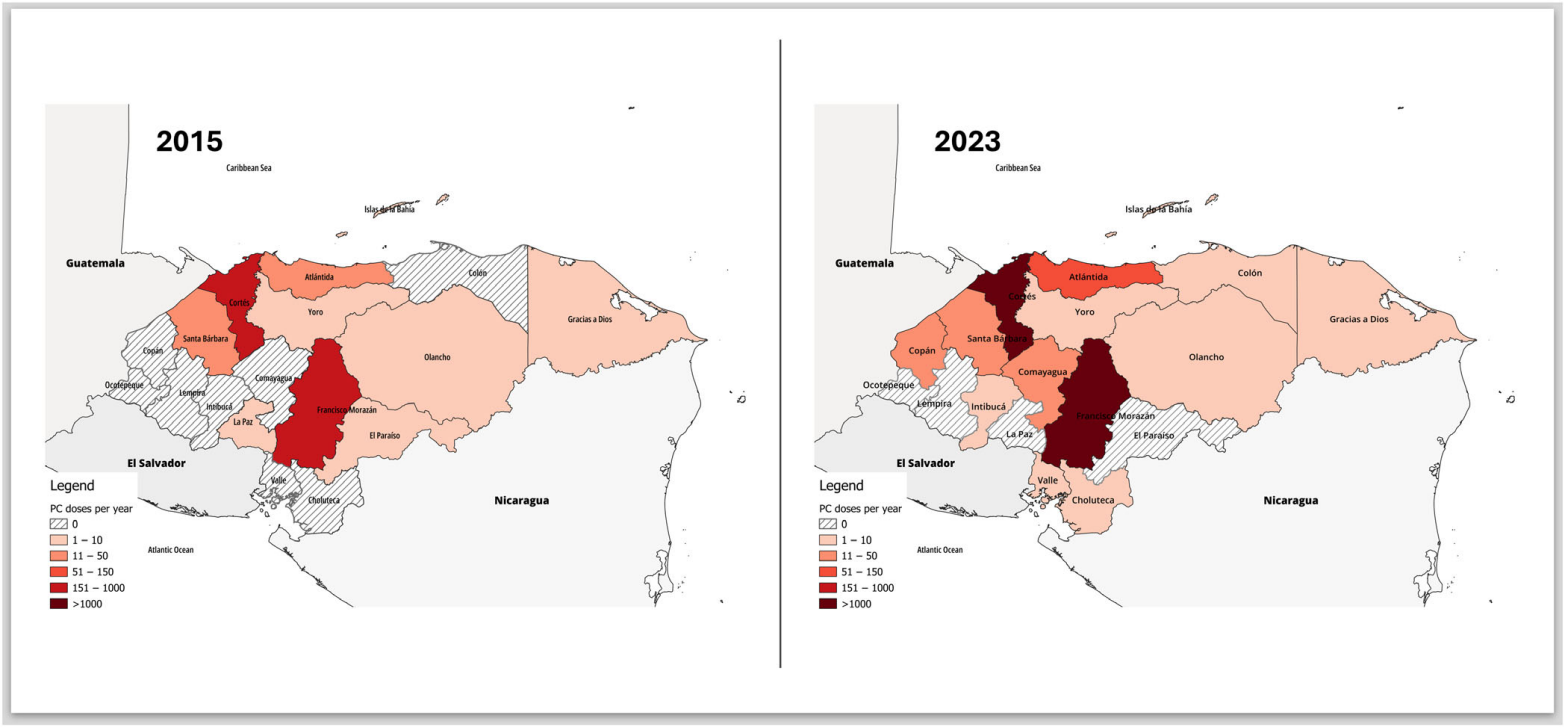
Evolution of national platelet distribution by region in Honduras, 2015 and 2023. PC, platelet concentrate.

Two regions (Francisco Morazán and Cortés) accounting for ~37% of the national population consumed 96% of PC doses in P1 and 88.3% in P2 (Table 3). In 2015, only 10 of 18 regions reported receiving one or more PRP doses per year. By 2023, 14 of 18 regions received at least 1 PR PC dose per year. Two low population regions (Lempira [~360,000 pop.] and Ocotepeque [~168,000 pop.]) received no PC doses in either period (Figure 3).

**TABLE 3:** Platelet Distribution by Region, Honduras, 2015–23.

	Period 1 (P1)				Period 2 (P2)					Mean by period		
Region	2015	2016	2017	2018	2019	2020	2021	2022	2023	P1	P2	Change (+/−)
Atlántida	23	19	41	44	259	215	254	161	142	32	206	+
Choluteca	0	0	0	0	0	5	0	0	7	0	2	+
Colón	0	0	1	0	6	30	1	1	8	0	9	+
Comayagua	0	6	3	8	7	15	4	1	24	4	10	+
Copán	0	2	4	0	1	7	8	30	23	2	14	+
Cortés	964	1122	1169	1215	1318	1367	1847	1579	1589	1117	1540	+
El Paraíso	1	0	1	1	12	10	31	8	0	1	12	+
Francisco Morazán	805	1036	886	661	1448	977	1556	1541	1275	847	1359	+
Gracias a Dios	2	0	0	0	5	3	11	0	1	0	4	+
Intibucá	0	1	0	0	0	0	3	0	1	0	1	+
Islas de la Bahía	3	1	7	2	4	6	13	4	8	3	7	+
La Paz	1	0	0	0	0	0	8	1	0	0	2	+
Lempira	0	0	0	0	0	0	0	0	0	0	0	nc
Ocotepeque	0	0	0	0	0	0	0	0	0	0	0	nc
Olancho	1	1	0	1	0	2	0	0	9	1	2	+
Santa Bárbara	38	32	46	4	5	28	4	8	35	30	16	−
Valle	0	0	0	0	0	0	1	1	2	0	1	+
Yoro	7	1	2	2	4	8	2	5	2	3	4	+
**National**	**1844**	**2221**	**2158**	**1938**	**3069**	**2673**	**3743**	**3340**	**3126**	**2040**	**3190**	

Abbreviation: nc, no change.

*Note*: The bold values represent the national totals of each year.

### Local validation of the amotosalen/UVA system and operational changes

**FIGURE 4:**
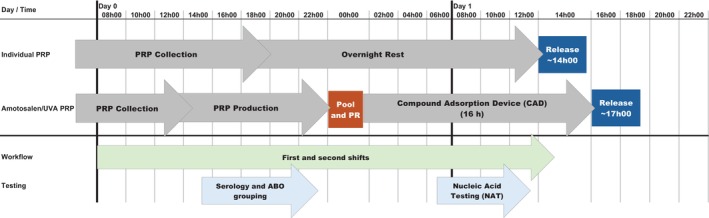
Honduran Red Cross (HRC) platelet production workflow and timeline before and after implementation of 100% pathogen‐reduced (PR) platelets. NAT, nucleic acid testing; PRP, platelet‐rich plasma.

The HRC validation study (*n* = 30 pools of six PRPs) confirmed that locally produced PR PCs met production parameters and acceptance criteria established by the manufacturer [19] and AABB Standards [20] (Table 4). Routine swirling and visual inspection procedures were also in place during both study periods. Certain workflow changes were required to accommodate the pathogen reduction process, which included PRP pooling, sterile docking to the INTERCEPT LV processing kit, illumination in the INTERCEPT Illuminator device and a 16‐h hold for the adsorption of residual amotosalen and photo‐products. These additional steps delayed PR PC release times by approximately 3 h compared with the routine process used to prepare individual PRP units; however, workflow changes had minimal impact on blood centre shift schedules and remained aligned with the HRC NAT and serology testing schedules (Figure 4).

**TABLE 4:** Results of HRC PR PC validation study, 2018.

*N* = 30 pools of six PRPs	Sample size	Volume (mL)^a^	Platelet concentration (×10^9^/L)^a^	Platelet dose (×10^11^)^a^	pH^a^, ^b^	RBC
Acceptance criteria (ranges)	Variable (20–60)	255–420	1050–2100	2.5–7.0	6.4–8.0	<4 × 10^6^/mL for 300–420 mL units or <4 × 10^5^/mL for 255–420 mL units
Mean (high/low daily range)	*N* = 30 pools of six PRPs	304 (280–320)	1245 (1020–1690)	3.8 (3.1–5.1)	7.3 (7.0–8.0)	All units within acceptance criteria
Median (IQR)		304 (8)	1188 (207)	3.6 (0.6)	7.0 (0.4)	

Abbreviations: IQR, interquartile range; PC, platelet concentrate; PR, pathogen‐reduced; PRP, platelet‐rich plasma; RBC, red blood cells.

^a^ Results of the validation study fell within acceptance ranges established by the manufacturer and AABB Standards for the production of pathogen‐reduced platelets.

^b^ pH values were measured daily post‐collection up to day 7.

### Cost–benefit analysis

As predicted by the McCullough et al. model, per dose costs were ~$100 higher in the pathogen reduction scenario compared with the baseline scenario ($74/dose baseline vs. $175/dose pathogen reduction). The increased costs were all associated with the addition of pooling sets and INTERCEPT Processing Sets. However, these increases were off‐set in the model by immediate savings achieved through the elimination of false‐positive bacterial screening results and savings associated with the avoidance of adding Zika virus screening for platelets. Additionally, the model captured savings associated with a reduction in the number of WB units required to produce PR PCs compared with the number of WB donations to produce single PRP units for a comparable number of ‘doses’ using the previous weight‐based dosing formula, reduced costs associated with TTIs and increased cost‐recovery linked to reduced waste. When these additional savings were accounted for, per dose costs only increased by $39. The avoidance of future costs associated with the potential introduction of gamma irradiation or CMV screening (neither of which were available in Honduras), and tests for certain emerging infectious diseases also contributed to a conclusion that pathogen reduction could be introduced and sustained in a cost‐neutral manner.

## DISCUSSION

The introduction of PR PCs with the amotosalen/UVA technology allowed the HRC to provide more PCs to patients in a wider geographical area with reduced waste, and no impact on overall whole blood collection requirements. The amotosalen/UVA pathogen reduction technology also added evidence‐based protection against bacterial [21, 22] and arboviral TTIs [23] without additional laboratory testing. For example, a 2023 meta‐analysis by Giménez‐Richarte et al. summarized pathogen inactivation studies showing the amotosalen/UVA technology achieved high levels of inactivation (≥4 log_10_, as recommended by the World Health Organization [24]) against Chikungunya (≥6.29 log_10_), Dengue (≥4.33 log_10_) and Zika (≥6.29 log_10_) viruses, three endemic arboviruses with epidemic potential in Latin America [25]. Likewise, in 2021 McDonald et al. demonstrated the amotosalen/UVA technology's capacity to achieve full inactivation of nine bacterial species commonly associated with TTBI through the end of 7‐days' storage [22].

As the first LMIC blood centre to adopt pathogen reduction as the standard of care for the majority of PCs produced, HRC has demonstrated the feasibility of implementing and sustaining pathogen reduction as a replacement for bacterial culture screening on a national scale. The HRC experience also shows how an LMIC may accrue additional safety benefits from pathogen reduction while avoiding future costs. Specifically, leukoreduction, gamma irradiation and CMV screening were not used in Honduras prior to pathogen reduction and have not been introduced since the adoption of pathogen reduction. In addition to reduced risk of viral, bacterial and protozoan threats, the amotosalen/UVA pathogen reduction may be used as an alternative to gamma irradiation for the prevention of TA‐GVHD and may replace CMV testing and leukoreduction for prevention of transfusion transmitted CMV infection [26, 27, 28]. The successful elimination of bacterial culture screening and irradiation with the introduction of amotosalen/UVA PR PCs for HSCT patients was described in 2019 by Sim et al. in a setting where leukoreduction was not used for platelets. A total of 33 patients received 76 non‐leukoreduced and non‐irradiated PR PCs without bacterial culture screening. Thirty‐one (31) control patients were transfused with 89 bacterial screened and irradiated PCs. The primary efficacy endpoint—1‐h corrected count increment (CCI)—was comparable in both cohorts which were followed for 100 days post‐transfusion. The rate of transfusion reactions (the primary safety endpoint) was reduced in the test cohort, but not significantly. The study also found that clinical refractoriness and refractory transfusions were significantly lower in the test cohort (*p* = 0.05 and *p* = 0.02, respectively), with no TA‐GVHD in either cohort and comparable rates of 100 day engraftment, mortality and infectious disease incidence in both cohorts [17].

Platelets remain a scarce commodity in Honduras, where <1 PC dose is available per 1000 population (by comparison up to eight PC doses are distributed per 1000 population per year in the United States [29]). PC distribution remains focused on urban areas, and barriers to PC distribution including inclement weather, poor roads and mountainous terrain were present in both P1 and P2. However, with up to 2 days' of additional shelf‐life, the introduction of pathogen reduction allowed a measurable increase in the availability of PCs in rural areas (~4% in P1 vs. ~12% in P2), where the Honduran Ministry of Health operates a network of general and basic hospitals [30].

While the true burden of cancer and other oncological conditions that may require acute or prophylactic platelet transfusion is unknown in Honduras, the incidence of multiple types of cancers (and need for supportive therapies) is projected to increase in LMICs in the coming decades [31]. Limited access to healthcare commodities and procedures and challenges with transportation have already been identified as key factors in delayed cancer diagnoses and treatments [32], abandonment of cancer therapy [33, 34] and poor cancer outcomes [35] in Honduras. Increased availability of PR PCs with a 7‐day shelf‐life in regional hospitals may help mitigate the impact of barriers to healthcare in rural areas [36, 37] and reduce pressure on urban hospitals where medical indications for transfusion compete with transfusion requirements for high levels of trauma due to violence and traffic accidents which may also require blood products [38, 39].

The introduction of amotosalen/UVA PR PCs in Honduras was accompanied by a major clinical shift in platelet dosing guidelines and an extension of PC shelf‐life from 5 to 7 days. The move from presumptive PC dosing based on patient weight to a standardized platelet dose per unit was well received by clinicians and has been shown in other LMIC settings to improve transfusion practice [40]. A clinical audit of platelet transfusion practice with standardized dosing is needed to understand the full clinical impact of this change in Honduras. A similar audit conducted over a 6‐year period in a large academic hospital in Mexico found that up to 25% of platelet transfusions were ‘inappropriate’ when assessed against British Society for Haematology guidelines, despite local training [41].

The HRC decision to adopt pathogen reduction for all PCs was driven by concerns about the limitations of culture screening as a guard against non‐bacterial TTIs [23] and made in the context of economic and logistical barriers to sustaining bacterial culture as a safety measure.

Since adopting pathogen reduction, HRC has worked with the amotosalen/UVA technology's manufacturer to limit year‐on‐year price increases and ensure a continuous supply of processing kits (since 2018 no PR stock‐outs have occurred). The introduction of pathogen reduction in Honduras also occurred during a period of substantial growth in government expenditure on health, driven in large part by the coronavirus disease 2019 (COVID‐19) pandemic. Between 2020 and 2023, public spending on hospital services increased >30%, with spending on materials and supplies rising by ~50% [42]. A fuller financial analysis is required to describe the impact of pathogen reduction within the overall growth of public sector spending on healthcare, validate savings accrued from the elimination of bacterial culture screening and estimate the avoidance of future costs associated with gamma irradiation, leukoreduction and CMV testing. Pending such an analysis real world cost savings or cost‐recovery opportunities may be inferred given the clinical and operational changes achieved in Honduras and cost modelling done in Canada and with a different pathogen reduction technology in another LMIC [43, 44]. Additional cost savings may include, for example the following:

1. Cost‐recovery from the higher number of transfusable units due to reduced waste.
2. Reduced healthcare costs associated with extended hospitalizations and specialty care associated with TTBI and emerging infectious diseases.

Additional research is also needed to assess patient safety trends in Honduras and compare the Honduran experience with positive clinical and safety trends described in other countries where the amotosalen/UVA technology has been adopted as the standard of care [45, 46, 47, 48].

This study is subject to several limitations. First, retrospective data may be subject to uncontrollable reporting biases, especially related to the distribution of blood components. It was beyond the scope of this study, for example, to track each ‘distributed’ unit to confirm transfusion. As a result, wastage rates presented here may underestimate levels of un‐used PC doses at the bedside. Second, the use of a mean adult weight (68 kg) to calculate the number of PRP PC doses in P1 may have produced under‐ or over‐estimates depending on the actual ratios of male and female adults transfused in a given region or hospital. This method also underestimates paediatric PC transfusions. While standardized dosing with six‐pool PRP PCs reduced the number of WB donations required to meet annual PC production quotas, the extent of this reduction is difficult to quantify. Third, individual patient outcomes were not available to assess the clinical benefit of increased access to PC transfusion in rural hospitals. Fourth, while clinicians reported preferring the new standard dosing system, data were not available to determine the number of PCs transfused per patient or other measures of appropriate PC transfusion practices in either period. Fifth, the economic analysis performed in 2017 does not account for inflation or other macroeconomic changes during the ensuing years; a fuller accounting of routine operating costs is warranted.

Despite these limitations, the HRC experience provides real world evidence of the feasibility of implementing and sustaining the amotosalen/UVA pathogen reduction technology with standardization of PC production and dosing, elimination of bacterial screening, avoidance of gamma irradiation and leukoreduction and 7‐day shelf‐life extension in an LMIC. Sustaining pathogen reduction as a routine blood centre process over a 5‐year period is particularly striking in Honduras, where per capita spending on health by the government is the lowest in the Latin America region. While public spending on health in Honduras has increased since 2010, Honduras continues to lag its neighbours in the region by a substantial margin [49]. This experience may provide insights and reassurance to other LMIC that pathogen reduction is not a cost‐prohibitive intervention when considered in the context of immediate cost savings, opportunities for cost‐recovery and the avoidance of future costs, especially those associated with the introduction of gamma irradiation and leukoreduction technologies or severe clinical outcomes in patients.

## CONFLICT OF INTEREST STATEMENT

J.P.P. is an employee and shareholder of Cerus Corporation.

## Data Availability

Data sharing not applicable to this article as no datasets were generated or analysed during the current study.
